# Burden of comorbidities in heart failure patients in Türkiye

**DOI:** 10.55730/1300-0144.5934

**Published:** 2024-05-07

**Authors:** Yüksel ÇAVUŞOĞLU, Selda MURAT, Anıl ŞAHİN, İnci Tuğçe ÇÖLLÜOĞLU, Dilek URAL, Mehmet Birhan YILMAZ, Sanem NALBANTGİL, Naim ATA, Mustafa Mahir ÜLGÜ, Şuayip BİRİNCİ, Emre DEMİR, Emine Arzu KANIK, Lale Dinç ASARCIKLI, Ahmet ÇELİK

**Affiliations:** 1 Department of Cardiology, Faculty of Medicine, Eskişehir Osmangazi University, Eskişehir, Turkiye; 2 Department of Cardiology, Faculty of Medicine, Sivas Cumhuriyet University, Sivas, Turkiye; 3 Department of Cardiology, Faculty of Medicine, Karabük University, Karabük, Turkiye; 4 Department of Cardiology, Faculty of Medicine, Koç University, İstanbul, Turkiye; 5 Department of Cardiology, Faculty of Medicine, Dokuz Eylül University, İzmir, Turkiye; 6 Department of Cardiology, Faculty of Medicine, Ege University, İzmir, Turkiye; 7 General Directorate of Health Information Systems, Ministry of Health, Ankara, Turkiye; 8 Ministry of Health, Ankara, Turkiye; 9 Department of Biostatistics, Faculty of Medicine, Mersin University, Mersin, Turkiye; 10 Department of Cardiology, Faculty of Medicine, Health Sciences University, Siyami Ersek Cardiovascular and Thoracic Surgery Training and Research Hospital, İstanbul, Turkiye; 11 Department of Cardiology, Faculty of Medicine, Mersin University, Mersin, Turkiye

**Keywords:** Comorbidity, comorbidity burden, electronic health records, heart failure

## Abstract

**Background/aim:**

Heart failure (HF) is associated with a wide range of comorbidities that negatively impact clinical outcomes and cause high economic burden. We aimed to evaluate the frequency and burden of comorbidities in HF patients in Türkiye and their relationships with patients’ demographic characteristics.

**Materials and methods:**

Based on ICD-10 codes in the national electronic database of the Turkish Ministry of Health covering the entire population of Türkiye (n = 85,279,553) from 1 January 2016 to 31 December 2022, data on the prevalence of comorbidities in HF patients were obtained. The frequency and burden of comorbidities were analyzed separately by age groups, sex, and socioeconomic status (SES).

**Results:**

Between 2016 and 2022, there were 2,722,151 patients (51.7% female) of all ages who were diagnosed with HF. In Türkiye, the most common comorbidities of HF patients were hypertension (HT) (97.6%), atherosclerotic cardiovascular disease (ASCVD) (84.9%), dyslipidemia (59.5%), anxiety disorder (48.1%), diabetes mellitus (DM) (45.2%), chronic obstructive pulmonary disease (COPD) (43.6%), anemia (40.6%), and atrial fibrillation (AF) (37.1%). Female patients had higher rates of anemia, DM, HT, and anxiety disorders, while male patients had higher rates of ASCVD, COPD, and dyslipidemia. The most common comorbidity in patients under 20 years of age was congenital heart disease (52.3%). More than 90% of HF patients had ≥2 comorbidities. HF patients with ≥5 comorbidities increased from 18.1% in the group aged 20–49 years to 38.3% in the group aged 50–79 years. Comorbidities were similar across SES groups.

**Conclusion:**

The most common comorbidities in cases of HF in Türkiye are HT, ASCVD, dyslipidemia, DM, COPD, anemia, and AF, respectively, and more than 90% of patients have ≥2 comorbidities. While ASCVD and dyslipidemia were more common in male patients, anemia, DM, and anxiety disorders were more common in female patients. The number of comorbid conditions increased with advanced age.

## 1. Introduction

Heart failure (HF) is an important community health problem that affects millions of people [1]. The prevalence and incidence of HF have been reported to continue to rise over the last decades [1]. Despite advances in the treatment of HF, prognosis is still poor, the rehospitalization rate is very high, and quality of life is worse [2,3]. HF is known to be associated with a wide range of cardiovascular and noncardiovascular comorbidities that have negative impacts on the clinical course of the disease, further impair both quality of life and clinical outcomes, add difficulties to the treatment of the clinical picture, and cause high economic burden in HF [2–4].

In clinical practice, the prevalence of comorbidities in HF has been reported to be very high, rising with more advanced disease, older age, and increased frailty. Some comorbid conditions might contribute to the development of HF, whereas others may lead to disease progression. Interactions between comorbidities and HF involve many pathways including neurohormonal overdrive, hemodynamic deterioration, inflammatory activation, oxidative stress, and endothelial dysfunction, although the details of these relationships are still under investigation. Furthermore, multiple comorbidities are common in patients with HF, with >85% of patients having ≥2 comorbid conditions [3,5]. Successful management of comorbidities is strongly recommended as a key component of the holistic care of patients with HF [2].

In Türkiye, data on the burden of comorbidities in patients with HF are primarily based on research conducted with previously published national registries and epidemiological studies, which generally have small study populations. The first study to provide information on HF prevalence in the adult Turkish population (age >35 years) was the HAPPY study conducted over 20 years ago. That study reported the absolute value of HF prevalence as 2.9% and also revealed that diabetes mellitus (DM), hypertension (HT), and chronic kidney disease (CKD) were more common comorbidities in HF patients [6]. The results of the TAKTIK registry for acute HF patients showed that coronary artery disease (CAD), HT, DM, and valvular heart disease were the most common comorbid conditions in acute HF patients in Türkiye [7]. In the SELFIE-TR registry that included both acute and chronic HF and patients with HF with reduced ejection fraction (HFrEF), HF with mildly reduced ejection fraction (HFmrEF), and HF with preserved ejection fraction (HFpEF), the most common comorbidities were reported as HT, CAD, DM, and chronic obstructive pulmonary disease (COPD) [8]. However, these national studies were generally conducted in academic centers and did not encompass all regions of Türkiye. Thus, they did not reflect population data for the whole country and they have important limitations.

In the recently published TRends-HF study, the nationwide HF patient population (n = 2,722,151) was evaluated using the national electronic database of the Turkish Ministry of Health based on International Statistical Classification of Diseases and Related Health Problems (ICD)-10 codes [9]. In the present study, we aimed to evaluate the burden of comorbidities in HF patients in Türkiye and their relationships with patients’ demographic characteristics based on data from the national electronic database of the Turkish Ministry of Health combined with the TRends-HF study.

## 2. Materials and methods

The design and analyses used in this study were published elsewhere among the details of the original article on the TRends-HF [9]. Briefly, this study was a nationwide population-based retrospective cohort study. The study protocol was approved and conducted in accordance with the Ministry of Health of Türkiye’s approval (Number 95741342-020). The design and procedure of the study were in accordance with the Declaration of Helsinki. This study used anonymized data from the Turkish Ministry of Health’s national electronic database. The database consisted of records for over 85 million citizens between 1 January 2016 and 31 December 2022.

The following ICD-10 codes were used to identify patients with HF: I50.0 (congestive HF), I50.1 (left ventricular dysfunction), I50.9 (HF, unspecified), I11.0 (hypertensive heart disease with congestive HF), I13.0 (hypertensive heart and chronic kidney disease with congestive HF), I13.2 (hypertensive heart and chronic kidney disease with congestive HF and renal failure), and I42.0 (dilated cardiomyopathy). Comorbidities including HT, atherosclerotic cardiovascular disease (ASCVD), dyslipidemia, anxiety disorders, DM, COPD, anemia, atrial fibrillation (AF), history of myocardial infarction, hypothyroidism, CKD, pulmonary embolism, iron deficiency, depression, ischemic stroke, hyperthyroidism, morbid obesity, hemorrhagic stroke, congenital heart disease (CHD), primary muscle disorders, and primary cardiac tumors were also obtained from the database using ICD-10 codes (Table 1). Since the Turkish Ministry of Health governs all relevant data, it is possible to cross-check the diagnoses with compatible medications after index diagnoses. The associations between comorbid conditions and sex and age groups were evaluated. To analyze the frequency of comorbidities among different age groups, we stratified the country’s population into four subpopulations based on the following age strata: (I) 0–19 years, (II) 20–49 years, (III) 50–79 years, and (IV) ≥80 years. The number of comorbidities per patient was also analyzed.

Categorical variables were expressed as numbers of cases and percentages (%). Data were stratified by sex and age groups. The association between comorbid conditions and sex was expressed as an odds ratio and confidence interval (CI) without any adjustment using the case–control study design model. Because of the size of the data, all comparisons could be statistically significant, but effect size and CI were calculated to decide whether data were clinically significant or not. Comorbidities based on sex were shown using a forest plot with odds ratios and CIs.

## 3. Results

In Türkiye, the most common comorbidity in HF patients is HT (97.6%). This was followed by ASCVD (84.9%), dyslipidemia (59.5%), DM (45.2%), COPD (43.6%), and anemia (40.6%). AF was observed in 37.1% of HF patients. Other comorbid conditions were previously shown in the Trends-HF study [9].

**Figure 1:**
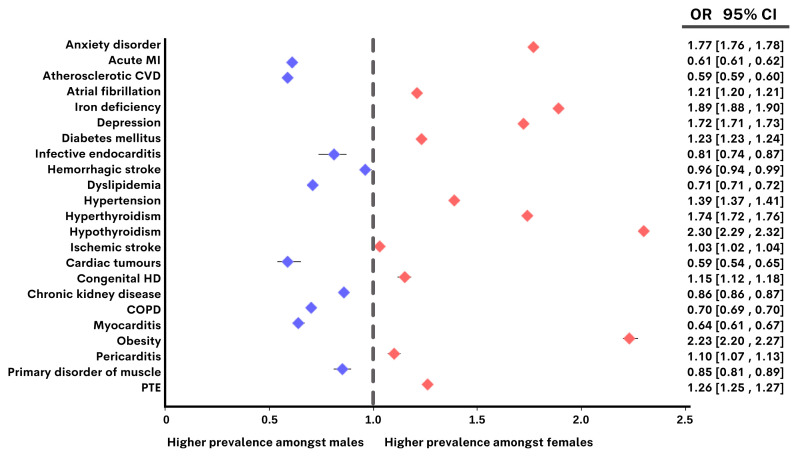
Unadjusted comparison of male and female patients according to the frequency of comorbidities in heart failure. COPD: Chronic obstructive pulmonary disease; HD: heart disease; MI: myocardial infarction; PTE: pulmonary embolism. Odds ratios of >1 indicate a greater prevalence of the condition among female patients, whereas odds ratios of <1 indicate a greater prevalence of the condition among male patients.

The most common comorbidity accompanying patients of both sexes with HF was HT. Our study shows that 97.2% of male patients and 97.9% of female patients had HT. Female patients had higher rates of anemia, DM, HT, hypothyroidism, pulmonary embolism, and anxiety disorders than male patients. The rate of AF was higher among female patients compared to male patients. It was also determined that 3.5% of patients with HF in Türkiye had morbid obesity, and this rate was higher among female patients compared to male patients (4.7% vs. 2.1%). On the other hand, male patients had higher rates of ASCVD, history of myocardial infarction, COPD, dyslipidemia, and CKD than female patients (Figure 1) [9].

The most common comorbidity in patients under 20 years of age was CHD (52.3%), while the most common comorbidities in the adult population were HT, ASCVD, hyperlipidemia, and DM. The most common comorbidities according to age groups are presented in Table 2.

The most common comorbidity in all socioeconomic status (SES) groups was HT, followed by ASCVD and dyslipidemia. Comorbidities were similar across SES groups. Comorbidities in HF patients according to SES and geographical regions were presented in previous articles [9,10].

When the comorbidity burden of the patients was evaluated, the rate of patients with no comorbid condition was less than 1% (0.8% for all patients, 0.9% for male patients, 0.7% for female patients). More than 90% of HF patients had ≥2 comorbid conditions, more than 80% of HF patients had ≥3, and nearly 60% of HF patients had ≥4 comorbidities. The rate of patients with ≥5 comorbidities was 35.9%. The comorbidity burden in all HF patients and by sex is presented in Table 3.

**Figure 2:**
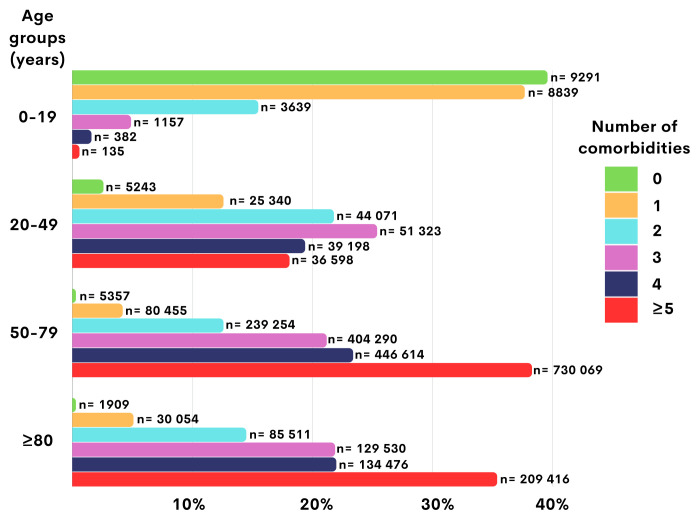
Comorbidity burden of patients with heart failure by age groups.

The number of comorbid conditions has been found to increase with advanced age. The rate of HF patients with no comorbidities was 39.6% in the age group of 0–19 years, while it was less than 1% in HF patients aged ≥50 years. More than 60% of patients in the group aged 20–49 years and more than 80% of patients in the group aged 50–79 years had ≥3 comorbid conditions. In the group aged >80 years, approximately one-third of the patients had ≥5 comorbidities. The percentage of HF patients with ≥5 comorbidities increased from 18.1% in the group aged 20–49 years to 38.3% in the group aged 50–79 years. The comorbidity burden by age groups is presented in Table 4 and Figure 2.

## 4. Discussion

This analysis based on data from the national electronic database of the Turkish Ministry of Health has revealed epidemiological data detailing the comorbidities accompanying HF in patients in the Turkish population. The most important findings of this study were as follows: First, the most common comorbidity in HF patients in Türkiye was HT and almost half of all HF patients had DM. Second, while the presence of ASCVD and dyslipidemia was more common in male patients, anemia, DM, and anxiety disorders were more common in female patients. Third, the most common comorbidity in patients under 20 years of age was CHD, and in other age groups (≥20 years), HT and ASCVD were the most common comorbid conditions accompanying HF. Fourth, comorbidities were similar across SES groups. Fifth, the rate of patients with no comorbidity was less than 1%, while the rate of patients with ≥5 comorbidities was approximately 35%.

Compared to data from Western countries, the rates of ASCVD, DM, dyslipidemia, COPD, and anemia were higher in the Turkish population of HF patients (Table 5) [9–12]. HT is the most frequently observed comorbid condition in Türkiye, as in Western countries [9–12]. In the GWTG-HF (“Get with The Guidelines-HF”) registry, which included approximately 40,000 HF patients, the rate of HT was 73.98% [11]. When we look at the frequency of DM, which is another important comorbidity in HF patients, the results of previous studies showed that the rate of DM varied between 31% and 45% [6,9–12]. Although the rate of ASCVD was higher in our study compared to Western countries, the previous studies reported that nearly one in every two HF patients had ASCVD.

Timely detection and optimal treatment of comorbid conditions that cause or accompany HF can prevent or slow the progression to HF. Additionally, correct management of comorbidities may reduce hospitalization and mortality rates [13]. Considering the data obtained mostly from Western countries, it is obvious that ASCVD can play a role in the etiology of HF and is also one of the most common comorbidities. In a study conducted in the United Kingdom, ASCVD was found to accompany HF in 49% of HF cases [14]. In a study including patients hospitalized for HF in the United States, this rate was 54% [14]. Our study showed that the coexistence of HF and ASCVD is much more serious in Türkiye. It has been observed that an average of 84.9% of people with HF have ASCVD. The high rate of ASCVD is noteworthy, especially in male HF patients. Based on the results of our study, we believe that more importance should be given to primary and secondary prevention in order to prevent HF. People at risk for ASCVD should be identified and lifestyle changes and medical treatment should be considered before HF develops.

Another important comorbidity that affects patient management and prognosis in HF patients is DM. In HF studies, the rate of diabetic patients was reported to be 26% in England, 21% in Norway, and 40% in the United States [13,15,16]. The data obtained in our study have shown that approximately half (45.7%) of HF patients in Türkiye have a diagnosis of DM. Although the presence of concomitant DM in HF patients in Türkiye is observed to be higher compared to Western countries, it is obvious that DM is a common problem around the world. There is a bidirectional interaction between HF and DM. It has been reported that patients with DM are 2–5 times more likely to develop HF than the general population [13]. In the management of these two chronic diseases, which are tightly intertwined, multidisciplinary health programs should be offered and access to guideline-directed optimal medical treatment should be facilitated.

Obesity is a growing public health problem around the world. The global prevalence of obesity almost tripled between 1975 and 2016, affecting most countries, including low-income and middle-income countries [17]. It is known that obesity may cause HF through direct or indirect pathophysiological mechanisms by causing cardiac hemodynamic, structural, and functional changes, or it may negatively affect the course of the disease in people diagnosed with HF [17]. When epidemiological studies were examined, it was observed that the prevalence of obesity accompanying HF was 32% in the United Kingdom, 2% in Denmark, and 6% in Germany [14,18–20]. In the HAPPY study conducted in Türkiye, the prevalence of obesity was 41.7% in all HF patients, 28.6% in HFrEF and HFmrEF patients, and 50.6% in HFpEF patients [6]. In our study, no definite data on the general prevalence of obesity could be obtained. On the other hand, the rate of morbid obesity was found to be 3.5%. It is obvious that policies and programs against obesity will reduce the risk of HF and improve the course of the disease.

Dyslipidemia is one of the common comorbidities of HF patients. In our study, it was observed that the frequency of dyslipidemia was increased in HF patients, especially after the age of 50. It has been reported that the frequency of dyslipidemia in HF patients reached 53% in the United States [15], while this rate was found to be 59.5% in our study. Although a direct relationship between dyslipidemia and HF has not been proven, it can be considered that dyslipidemia has an indirect effect on HF due to the relationship between ASCVD and dyslipidemia. Indeed, in our study, the prevalence of both dyslipidemia and ASCVD was found to be higher in male patients than in female patients. It should be taken into consideration that correct management of dyslipidemia may prevent the emergence of many diseases, especially ASCVD and HF.

AF is the most common chronic rhythm disorder in the course of HF, and the presence of AF in HF patients is closely associated with poor prognosis. Data from Western countries show that HF is accompanied by AF at rates ranging from 10% to 50% [21,22]. In this study, the AF rate in HF patients in Türkiye was found to be 37.1%. In the HAPPY study, the AF rate was reported as 10.6%, which is almost one-third the rate obtained in our study [21]. The fact that the rate is higher in female patients than in male patients (39.2% vs. 34.8%) may be due to the higher frequency of HFpEF in women. Since HF phenotyping could not be performed in our study, the relationship between AF rates and HF types could not be evaluated. However, considering the close association of HF and AF, the management of these two conditions together is very important.

Anemia and iron deficiency are common comorbidities in all age groups in HF patients in Türkiye. Studies conducted in Spain and Denmark reported that the association of anemia with HF was about 10%–12% [19,23]. The incidence of iron deficiency in patients with symptomatic HF has been reported to be up to 50% [24]. Iron deficiency is associated with worse functional capacity, lower quality of life, and increased mortality in HF. In Türkiye, the prevalence of anemia and iron deficiency in HF patients is 40.6% and 32.5%, respectively, and the rates of both anemia and iron deficiency are higher in women than in men. In addition, the most common comorbidity after CHD in HF patients under 20 years of age was anemia.

Another common comorbidity in HF patients is depression. Depression is associated with poor prognosis in HF [13]. In our study, anxiety disorders and depression were found at high rates in HF patients. This was especially noticeable in female patients. In a Swedish cohort, psychiatric disorders were reported at a rate of 13% in HF patients, and in a Danish study, it was reported that 20% of people with HF used antidepressant treatments [19,25]. In our study, the rate of anxiety disorders in HF patients in Türkiye was found to be almost 50%, and the rate of depression was approximately 30%. These results indicate the necessity of developing healthcare policies to manage anxiety and depression in HF cases. The current European Society of Cardiology HF guidelines also recommend encouraging patients to seek help for psychological problems such as depressive symptoms, anxiety, or low mood that may occur during the course of HF as part of patient education and self-care [2].

In patients with HF, the burden of comorbidities is important in addition to the individual assessment of comorbidities. Noncardiovascular comorbidity characteristics in hospitalized HF patients were investigated by evaluating data from nearly 200,000 patients in the GWTG-HF registry. In this registry, the prevalence rates of 0, 1, 2, and ≥3 noncardiovascular comorbidities were 18%, 30%, 27%, and 25%, respectively. It was also shown that patients admitted to the hospital for HF had increasing numbers of noncardiovascular comorbidities over time, leading to worse outcomes [26]. HF patients have to cope with a heavy burden of noncardiovascular comorbidities that can increase the risk of mortality and reduce quality of life. Among US Medicare beneficiaries, 40% of HF patients are reported to have >5 noncardiovascular comorbidities [27]. The European Society of Cardiology HF Pilot Study reported that approximately 75% of outpatients with chronic HF had at least one noncardiovascular comorbidity. Additionally, increasing numbers of comorbidities have been associated with an increase in the risk of mortality [28]. In our study, regardless of sex, more than 90% of patients had ≥2 comorbid conditions and almost two-thirds of patients had ≥4 comorbidities. When the comorbidity burden was examined by age, it was observed that it increased with age. Strategies aimed at reducing the increasing burden of noncardiovascular comorbidities are necessary to improve outcomes and should be included in HF patient management [26].

Finally, the comorbidity features of HF patients under 20 years of age were also examined in this study. In the current literature, data on HF patients under the age of 20 are quite limited. It is thought that the results obtained from the data of more than 23,000 patients in this age group in the present study will contribute to the development of health strategies. When the most common comorbidities in HF patients were examined according to age groups, it was seen that CHD is the leading problem in patients under 20 years of age. CHD is present in 69.5% of the HF population aged 0–9 years. Accurate and early diagnosis of CHD, including in the antenatal period, will reduce the risk of progression to HF.

As has been stated in the original published article on the TRends-HF study [9], the main limitation of that study was the lack of data on ejection fraction (EF). Similarly, the relationship between comorbidities and HF types could not be evaluated in the present study because EF values could not be obtained due to system-based shortcomings; therefore, we were unable to analyze patient data according to EF values. Additionally, we did not have access to the full data of all 85 million citizens, and so we could not compare the data of the HF population with the non-HF population. Moreover, the diagnosis of HF relied on ICD-10 codes input by clinicians, raising uncertainty about the accuracy of diagnoses for all patients. Although cross-referencing diagnoses with corresponding medications for index diagnoses was feasible, the presence of undiagnosed HF patients or those diagnosed but not recorded in the database remains a potential limitation.

## 5. Conclusion

Comorbidities are quite common in HF patients, with differences seen according to age groups and sex. The management of comorbidities is an important part of optimal HF management and the basis of a holistic approach. Our study is the most comprehensive study to date examining comorbidities in the whole HF patient population in Türkiye. In light of the data in our study, comorbid conditions that are common in the HF population in Türkiye should be taken into consideration in patient management and healthcare policies. It should be kept in mind that optimal and timely treatment of these comorbidities may improve clinical outcomes and the burden of the disease.

## Figures and Tables

**Table 1:** ICD-10 codes used in the diagnosis of heart failure and the determination of comorbidities.

	ICD-10 codes
Diagnosis	
Heart failure	I50.0, I50.1, I50.9, I11.0, I13.0, I13.2, I42.0
Comorbidities	
Hypertension	I10
Atherosclerotic cardiovascular disease	I25, I70
Dyslipidemia	E78
Anxiety disorder	F41
Diabetes mellitus	E10, E11, E13, E14
Chronic obstructive pulmonary disease	J44
Anemia	D63, D64
Atrial fibrillation	I48
Iron deficiency anemia	D50
Depression	F31, F32, F33, F34.1, F06.3
Acute myocardial infarction	I21
Hypothyroidism	E03
Chronic kidney disease	N18
Pulmonary embolism	I26
Ischemic stroke	G45, I63
Hyperthyroidism	E05
Obesity	E66
Congenital heart disease	Q21, Q22, Q23, Q25
Pericarditis	I30, I32, I39
Myocarditis	I40, I41
Primary muscles disorders	G71
Infective endocarditis	I33, I38

**Table 2:** Most common comorbidities in cases of heart failure by age groups.

Age groups	Common comorbidities	n (%)
0–19 years (n = 23,443)	CHD	12,258 (52.3)
	Anemia	8362 (35.7)
	Iron deficiency	7033 (30)
	Hypothyroidism	2870 (12.2)
20–49 years (n = 201,773)	Hypertension	184,805 (91.6)
	ASCVD	151,408 (75)
	Dyslipidemia	100,620 (49.9)
	Anxiety disorders	89,935 (44.6)
50–79 years (n = 1,906,039)	Hypertension	1,879,950 (98.6)
	ASCVD	1,669,048 (87.6)
	Dyslipidemia	1,255,137 (65.9)
	Diabetes mellitus	961,251 (50.4)
≥80 years (n = 590,896)	Hypertension	581,562 (98.4)
	ASCVD	487,832 (82.6)
	COPD	303,952 (54.2)
	Anxiety disorders	303,277 (51.3)

ASCVD: Atherosclerotic cardiovascular disease; CHD: congenital heart disease; COPD: chronic obstructive pulmonary disease.

**Table 3:** Comorbidity burden in heart failure patients by sex.

Comorbidity burden*	All patients (n = 2,722,151)	Male (n = 1,314,224)	Female (n = 1,407,927)
No comorbidities	21,800 (0.8%)	12,350 (0.9%)	9450 (0.7%)
1 comorbidity	144,688 (5.3%)	68,949 (5.2%)	75,739 (5.4%)
2 comorbidities	372,475 (13.7%)	182,472 (13.9%)	190,003 (13.5%)
3 comorbidities	586,300 (21.5%)	294,217 (22.4%)	292,083 (20.7%)
4 comorbidities	620,670 (22.8%)	303,362 (23.1%)	317,308 (22.5%)
≥5 comorbidities	976,218 (35.9%)	452,874 (34.5%)	523,344 (37.2%)

* While calculating the comorbidity burden, hypertension, atherosclerotic cardiovascular disease, diabetes mellitus, hyperlipidemia, chronic kidney disease, chronic obstructive pulmonary disease, atrial fibrillation, anemia/iron deficiency, stroke, and depression were taken into account.

**Table 4:** Comorbidity burden in heart failure patients by age groups.

Comorbidity burden*	0–19 years (n = 23,443)	20–49 years (n = 201,773)	50–79 years (n = 1,906,039)	≥80 years (n = 590,896)
No comorbidities	9291 (39.6%)	5243 (2.6%)	5357 (0.3%)	1909 (0.3%)
1 comorbidity	8839 (37.7%)	25,340 (12.6%)	80,455 (4.2%)	30,054 (5.1%)
2 comorbidities	3639 (15.5%)	44,071 (21.8%)	239,254 (12.6%)	85,511 (14.5%)
3 comorbidities	1157 (4.9%)	51,323 (25.4%)	404,290 (21.2%)	129,530 (21.9%)
4 comorbidities	382 (1.6%)	39,198 (19.4%)	446,614 (23.4%)	134,476 (22.8%)
≥5 comorbidities	135 (0.6%)	36,598 (18.1%)	730,069 (38.3%)	209,416 (35.4%)

* While calculating the comorbidity burden, hypertension, atherosclerotic cardiovascular disease, diabetes mellitus, hyperlipidemia, chronic kidney disease, chronic obstructive pulmonary disease, atrial fibrillation, anemia/iron deficiency, stroke, and depression were taken into account.

**Table 5:** Comparison of comorbidity rates in epidemiological heart failure studies.

	TRends-HF (n = 2,722,151)	ESC-HF Long-Term Registry		GWTG-HF (n = 39,982)
		(HHF n =5039)	(CHF n =7401)	
Hypertension	97.6%	64.5%	58.2%	73.98%
ASCVD	84.9%	54.0%	43.0%	50.59%
Dyslipidemia	59.5%	-	-	42.05%
Diabetes mellitus	45.2%	38.9%	31.8%	38.82%
COPD	43.6%	20.2%	13.8%	27.61%*
Anemia	40.6%	-	-	17.55%
Atrial fibrillation	37.1%	44.0%	37.6%	36.78%
CKD	17.7%	26.4%	18.2%	18.51%**

ASCVD: Atherosclerotic cardiovascular disease; CHF: chronic heart failure, CKD: chronic kidney disease; COPD: chronic obstructive pulmonary disease; GWTG-HF: Get with the Guidelines–HF Registry; HHF: hospitalized heart failure.

* COPD or asthma.

** In the GWTG-HF registry, chronic renal insufficiency was defined as serum creatinine of >2.0 mg/dL.
